# Ultra‐processed food consumption and obesity among children and adolescents in China—Findings from China Health and Nutrition Survey

**DOI:** 10.1111/ijpo.70012

**Published:** 2025-03-12

**Authors:** Ming Li, Zumin Shi

**Affiliations:** ^1^ Faculty of Medicine University of Queensland Brisbane Queensland Australia; ^2^ Human Nutrition Department, College of Health Sciences, QU Health Qatar University Doha Qatar

**Keywords:** adolescents, children, China, long‐term consumption, obesity, overweight, ultra‐processed food

## Abstract

**Background:**

Children and adolescents are increasingly exposed to processed food in China, however, its association with obesity has not been investigated.

**Objectives:**

To assess the consumption of ultra‐processed food (UPF) and its association with overweight/obesity among children and adolescents in China.

**Methods:**

A total of 3437 children and adolescents aged 6–18 years, participating at least twice in the China Nutrition and Health Survey, were included. Food intake was collected using a 3‐day 24‐h dietary recall method at home visits. Body weight, height and waist circumference (WC) were measured during the survey. UPF was defined by food process levels using NOVA classification. Overweight/obesity was defined by the international age‐ and sex‐specific BMI and WC cut‐offs. The association between UPF consumption and overweight/obesity was assessed using mixed effect logistic regression analyses adjusted for socio‐demographic, economic, behavioural, dietary and health factors.

**Results:**

The mean daily UPF consumption of the study population (mean age 9.3 years) increased from 9.7 in 1997 to 60.0 grams in 2011. The adjusted odds ratios (OR) (95% CI) for overweight/obesity (using BMI) for UPF consumption of 0, 1–49, 50–99 and ≥ 100 g/day were 1.00, 1.38 (0.98–1.94), 2.01 (1.25–3.24) and 1.53 (0.82–2.86), respectively (*p*‐trend =0.013). Similarly, the corresponding adjusted ORs (95% CI) for central obesity (using WC) were 1.00, 1.84 (1.30–2.60), 2.13 (1.30–3.48) and 2.15 (1.14–4.05) (*p*‐trend<0.001).

**Conclusions:**

Higher long‐term UPF consumption was associated with an increased risk of overweight/obesity among children and adolescents in China.

**Abbreviations**
CNHS: China Nutrition and Health Survey
IOTF: International Obesity Task Force
MET: metabolic equivalent of task
OR: odds ratios
UPF: ultra‐processed food
WC: waist circumference

## 1 INTRODUCTION

Overweight and obesity in children and adolescents have become a major public health concern in China. A national report shows the prevalence of obesity in school‐aged children nearly doubled from 15.5% in 2010 to 29.4% in 2020.^1^ Previous findings have established that overweight/obesity in children and adolescents significantly increases the risk of early onset of chronic diseases, including diabetes, hypertension, metabolic syndrome and negatively impacts mental health and well‐being.^2^ The growing burden of overweight and obesity in children and adolescents could be driven by economic developments, socio‐cultural norms and policies that have shaped individual‐level risk factors through urbanization, urban planning, built environments and food systems and environments.^3^ Substantial changes in dietary patterns have occurred in Chinese populations, with increased consumption of animal‐sourced foods, refined grains, highly processed, high‐sugar and high‐fat foods. This modern dietary pattern has been associated with cardiometabolic health risk in adults.^4^ Our earlier study has also shown that higher consumption of soft drinks and energy‐dense fast foods are positively associated with overweight and obesity among school‐aged adolescents,^5^ while physical inactivity is also associated with the increased risk of obesity.^6^

Another remarkable change in the past decades in China is the increased purchasing and consumption of processed and semi‐processed foods. Contributing to this change are improved transportation, longer shelf life and improved taste, making these products more accessible in supermarkets. Ultra‐processed foods (UPF) are convenient, highly palatable and profitable foods made from inexpensive ingredients and refined extracts from foods using a series of industrial formulations and processes.^7^ UPF are the fourth food group based on NOVA (not an acronym) classification, a holistic approach that conceptually shifts nutrition science by not only considering the physical extent of processing but also the purpose of processing.^7^ Using data from the China Nutrition and Health Survey (CNHS), we have reported that the mean UPF consumption increased from 12.0 grams (g) in 1997 to 41.5 g in 2011, with the corresponding proportion of UPF in the daily diet increasing from 1.0% to 3.6% among 12,451 adults aged >20 years in the same period.^8^ Higher UPF consumption is associated with increased risk of overweight and obesity, diabetes and hypertension in Chinese adults,^8^, ^9^, ^10^ consistent with the synthesis of 48 studies from Europe and the Americas which identify plausible underlying mechanisms.^11^ Several mechanisms have been proposed to explain the link between UPF and obesity, such as overconsumption. This includes their nutrient and energy content, displacement of healthy food groups, matrix degradation, altered texture and dysregulation of mechanisms of weight regulation.^7^

Evidence on UPF consumption and its relationship with overweight/obesity in children and adolescents is limited and the findings to date are inconsistent. For example, a 3‐year follow‐up study among Brazilian adolescents reported no association between UPF intake and weight status, although higher UPF consumption was linked with poorer diet quality.^12^ Conversely, several longitudinal studies in children from Europe and the Americas have shown that higher consumption of UPF predicts higher BMI or adiposity over follow‐up periods ranging from 1 to 10 years.^13^, ^14^, ^15^, ^16^, ^17^, ^18^, ^19^ To date, there has been no report in China examining the relationship between UPF consumption and overweight/obesity in children and adolescents. The aim of this study is to address this knowledge gap by analysing data from children and adolescents aged 6–18 years who participated in the CNHS between 1997 and 2011.

## 2 METHODS

### 2.1 Study design and sample

This study assesses UPF consumption and its association with overweight and obesity using repeated measurements of dietary intake and anthropometry from 1997 to 2011, based on CHNS data.

**FIGURE 1:**
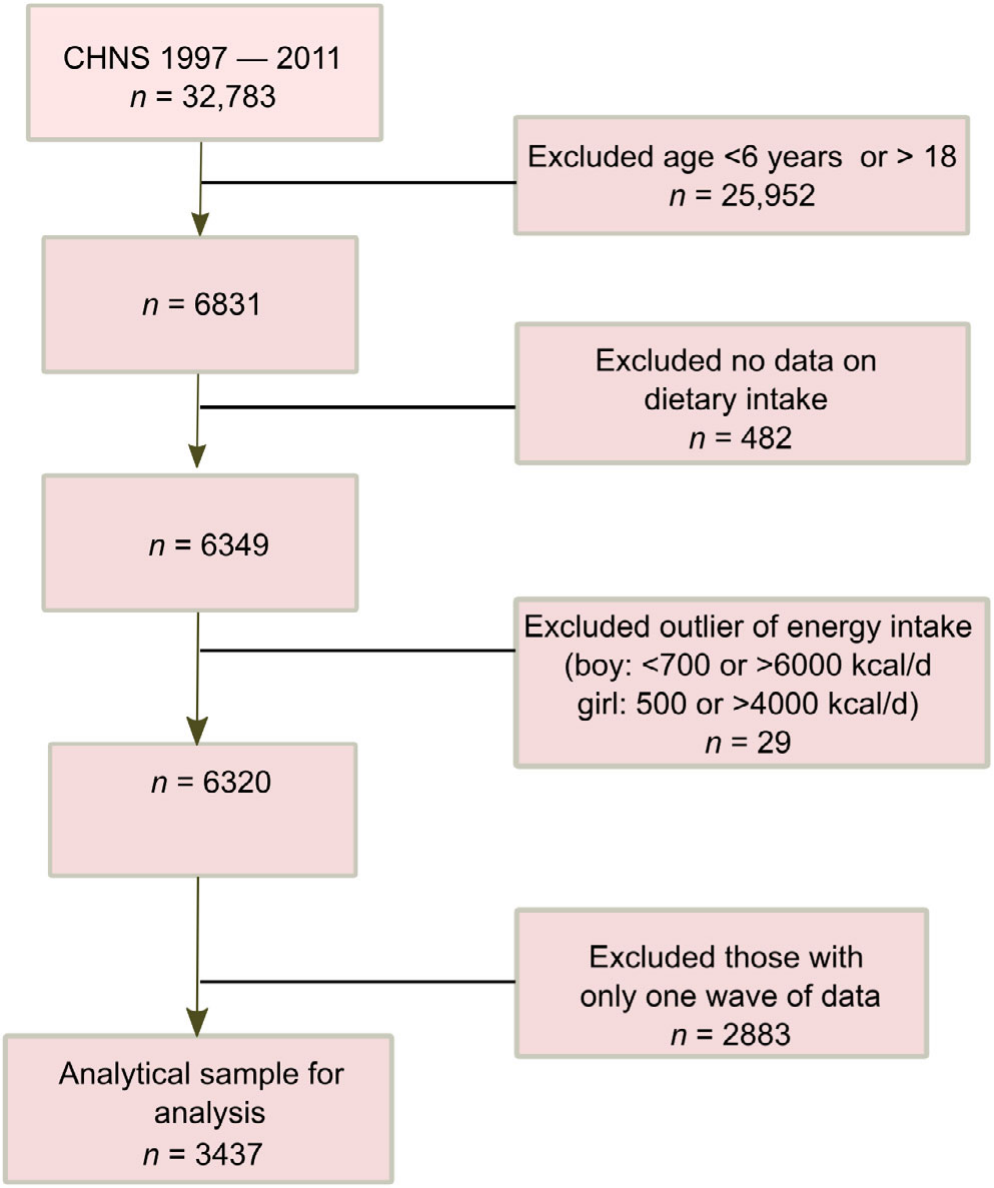
Sample flow chart of participants attending CHNS 1997–2011.

The CHNS is an ongoing, household‐based, open cohort study conducted in nine provinces in China,^20^ employing a multistage random‐cluster sampling process in both urban and rural areas. The study has completed 11 waves of data collection (1989, 1991, 1993, 1997, 2000, 2004, 2006, 2009, 2011, 2015 and 2018). All members in the randomly selected households are invited to join and are free to repeat or leave the subsequent surveys, therefore, the participation round ranges between 1 and 11. Over 60% of participants completed all survey rounds during the study period 1989–2006, and more than 80% completed at least two rounds within the same time frame.^20^ UPF intake was rare and lacked variation prior to the 1997 surveys. Currently, the 2015 and 2018 survey data are not publicly available. Ultimately, this study included 3437 children and adolescents who met the following inclusion criteria (Figure 1): aged 6–18 years; participated in at least two nutrition surveys during 1997–2011; had dietary intake and anthropometric measures of weight, height and waist circumference (WC); and had plausible energy intake (males: 700–6000 kcal/day, females: 500–4000 kcal/day).

The study was conducted in accordance with the guidelines of the Declaration of Helsinki. All procedures involving research participants were approved by the institutional review boards of the University of North Carolina (USA) and the National Institute of Nutrition and Food Safety (China). Written informed consent was obtained from all participants and/or their guardians.

### 2.2 Outcome variable: Overweight and obesity

Anthropometric data were collected by trained health workers using standardized protocols during household visits at each survey. Height was measured without shoes to the nearest 0.2 cm using a portable stadiometer. Weight was measured without shoes and in light clothing to the nearest 0.1 kg using a calibrated beam scale. WC was measured to the nearest 0.1 cm using a Seca tape measure at the midpoint between the lowest rib margin and the iliac crest. BMI was calculated as weight (kg) divided by height (m) squared. Overweight/obesity was defined using the International Obesity Task Force (IOTF) age‐sex‐specific BMI cut‐offs,^21^ and the WC references generated by applying the same IOTF method.^22^

### 2.3 Exposure variable: UPF consumption

At each survey, individual dietary intake data were collected by a trained investigator using a 24‐h dietary recall over three consecutive days during home visits.^20^ The household adult reported the child's food intake, with the child around to remind them. In addition, all foods consumed during the survey period were weighed and assigned to each household member. Specifically, foods and condiments in the home inventory, foods purchased from markets or picked from gardens and food waste were weighed and recorded by interviewers at the beginning and end of the three‐day survey period. The types and amount of food, the type of meal and the place of consumption for a participant were derived from both dietary recall and the records kept by the individual. Cooking oil and condiments consumption for everyone in the household was estimated using individual energy‐weighted intake. A detailed description of the dietary measurement has been published previously.^23^ The food data were recoded and converted to nutrient intake using updated food composition tables.^24^, ^25^, ^26^ Since 1997, around 3000 different food items have been categorized into four groups based on the NOVA classification.^7^ Any uncertain food item(s) were discussed, and consensus was reached on their processing status. For example, the fruity or milky drinks containing sweeteners, preservatives and other additives were classified as UPF.

### 2.4 Covariates

Socio‐demographic and lifestyle factors were collected at each survey using a structured questionnaire. Socio‐economic status: education (low: illiterate/primary school; medium: junior middle school; high: high middle school or higher), per capita annual family income (recoded into tertiles as low, medium and high), urbanization levels (recoded into tertiles as low, medium and high).

Sleep duration was recorded as 6, 7–9 and more than 9 h per day using data collected since 2004. Physical activity level (metabolic equivalent of task (MET)) was estimated based on self‐reported activities (including domestic, transportation and leisure‐time physical activities) and duration using a Compendium of Physical Activities. There was no smoker and only 13 participants reported alcohol‐drinking, so these variables were excluded from the analysis.

Two dietary patterns (traditional and modern) were identified using a method previously applied in the adult population. The traditional pattern was characterized by high intake of rice, meat and vegetables, while the modern pattern was highly correlated with fast food, milk and deep‐fried food.^4^

### 2.5 Statistical analysis

UPF intake was categorized into four levels: non‐consumers, 1–49, 50–99 and ≥100 g/day. These cut‐offs were chosen based on the serving size in the context of Chinese food, where a serving is approximately 50 g (Liang). Baseline characteristics were presented and compared by baseline UPF intake levels using ANOVA for continuous measures or Chi‐square tests for categorical ones. The age‐ and sex‐adjusted UPF consumption, BMI, WC and the prevalence of overweight/obesity during the study period were estimated using marginal linear regression and logistic regressions.

The association between UPF consumption and overweight/obesity defined by both BMI and WC was assessed with mixed effect logistic regression analysis. Unadjusted and adjusted odds ratios (OR) and 95% confidence intervals (95% CI) of the fixed part of the mixed effect models were reported, accounting for within‐person variation as the random part. Several models were used: an unadjusted model; Model 1 adjusted for age, sex and energy intake by using the following STATA command: melogit overweight i.upf_levels age i.sex energy_intake ||id, or; Model 2 further adjusted for socio‐economic status (income, urbanization and parental education), fat intake, physical activity; Model 3 further adjusted for dietary patterns.

The interaction between UPF intake and socio‐demographic factors (age, sex, residence) on overweight/obesity was assessed by introducing a product term in the regression model. All analyses were performed using STATA 18.0 (Stata Corporation, College Station). Statistical significance was set at *p* < 0.05 (two‐sided).

## 3 RESULTS

### 3.1 Population characteristics at entry

A total of 3437 children and adolescents were included in this analysis. Among them, 52.4% entered in 1997, 15.9% in 2000, 15.0% in 2004, 7.2% in 2006 and 9.5% in 2009. At entry, 85.3% were aged 6–12 years. Overall, 53.5% were males, and 76.3% were residing in rural areas. The prevalence of overweight/obesity and central obesity was 9.7% and 5.3%, respectively. The mean daily intake (g/day) of carbohydrate, protein and fat was 262.9 (SD 95.1), 52.3 (SD 19.5) and 51.4 (SD 29.8). The daily mean intake of fruit and fresh vegetables (g/day) was 24.8 (SD 76.6) and 210.4 (SD 188.3). The daily mean UPF intake was 15.8 g (SD 44.6). Smoking and alcohol drinking were nearly not existing. Approximately 43% of the participants reported a daily mean sleeping time >9 h in the 2004–2009 survey.

### 3.2 The consumption of UPF during 1997–2011

**FIGURE 2:**
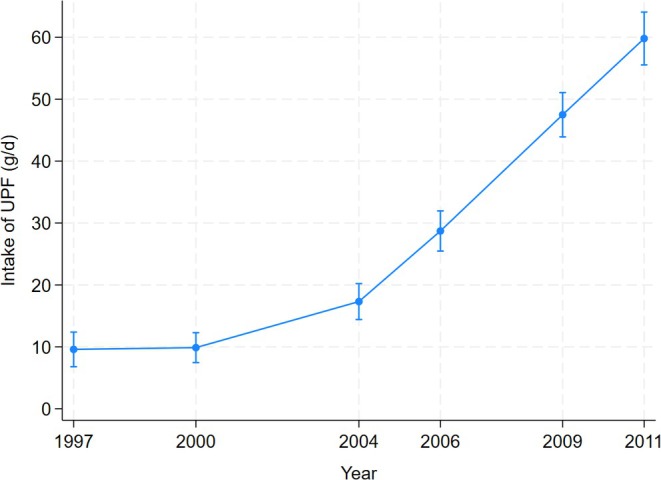
Age‐ and sex‐adjusted mean per capita UPF consumption (g/day) among children and adolescents attending CHNS in 1997–2011. UPF consumption estimated from linear regression adjusted for age and sex.

The overall age‐ and sex‐adjusted mean per capita consumption of UPF increased from 10 g/d in 1997 to 47 g/d in 2009 and further to 60 g/day in 2011, with a sharp rise since 2004 (Figure 2). The most 10 frequently reported UPF items were pre‐packed Baozi, bread, instant noodles, cake, sausage, pre‐packed Jiaozi, Coke, cookie, yogurt‐flavoured beverage and ice lolly. Among the 3437 children and adolescents participating in the baseline surveys, 77% of them did not have any UPF, 12% had 1–49 g/day, 6.3% had 50–99 g/day and 5.0% consumed a daily UPF ≥ 100 g/day. Of the 173 participants having UPF ≥ 100 g/day, 38 entered the surveys in 1997 (22%) and 67 in 2009 (39%).

At entry, UPF consumption was significantly higher in: children than in adolescents; those from higher‐income households; those living in urban than in rural areas (*p* < 0.05). Children and adolescents having UPF ≥ 100 g/d also had a higher intake of energy, fat, protein, carbohydrate and fruits but a lower intake of vegetables, and a higher modern dietary pattern score. Significantly more children and adolescents having UPF ≥ 100 g/day had shorter sleep time (6–9 h/day) compared to those with less UPF consumption (*p* < 0.001) (≥9 h/day). The mean physical activity MET score was higher in those with higher UPF intake compared to those with low intake (*p* < 0.001) (Table 1).

**TABLE 1:** Baseline sample characteristics by UPF intake among children and adolescents aged 6–18 years attending CHNS 1997–2011 (*n* = 3437).

	UPF intake (g/day)					
	None	1–49	50–99	≥100	Total	*p* value
*N*	2628 (76.5)	421 (12.2)	215 (6.3)	173 (5.0)	3437 (100.0)	
Year of entry (%)						
1997	1459 (55.5)	225 (53.4)	78 (36.3)	38 (22.0)	1800 (52.4)	<0.001
2000	426 (16.2)	60 (14.3)	36 (16.7)	23 (13.3)	545 (15.9)	
2004	392 (14.9)	56 (13.3)	42 (19.5)	27 (15.6)	517 (15.0)	
2006	181 (6.9)	29 (6.9)	20 (9.3)	18 (10.4)	248 (7.2)	
2009	170 (6.5)	51 (12.1)	39 (18.1)	67 (38.7)	327 (9.5)	
Age (years)	9.4 (2.6)	9.2 (2.6)	9.1 (2.5)	8.8 (2.5)	9.3 (2.6)	0.020
Age group (%)						
6–12	2222 (84.6)	368 (87.4)	188 (87.4)	155 (89.6)	2933 (85.3)	0.111
13–18	406 (15.4)	53 (12.6)	27 (12.6)	18 (10.4)	504 (14.7)	
Sex (%)						
Male	1419 (54.0)	209 (49.6)	112 (52.1)	99 (57.2)	1839 (53.5)	0.271
Female	1209 (46.0)	212 (50.4)	103 (47.9)	74 (42.8)	1598 (46.5)	
Income (%)						
Low	1084 (41.8)	141 (34.2)	68 (32.1)	41 (24.3)	1334 (39.4)	<0.001
Medium	892 (34.4)	152 (36.9)	77 (36.3)	53 (31.4)	1174 (34.7)	
High	619 (23.9)	119 (28.9)	67 (31.6)	75 (44.4)	880 (26.0)	
Education (%)						
Primary school	1947 (84.7)	326 (87.2)	164 (88.2)	133 (87.5)	2570 (85.3)	0.285
Junior high	335 (14.6)	43 (11.5)	22 (11.8)	17 (11.2)	417 (13.8)	
Senior high	18 (0.8)	5 (1.3)	0 (0.0)	2 (1.3)	25 (0.8)	
Residence (%)						
Rural	2095 (79.7)	289 (68.6)	134 (62.3)	103 (59.5)	2621 (76.3)	<0.001
Urban	533 (20.3)	132 (31.4)	81 (37.7)	70 (40.5)	816 (23.7)	
BMI (kg/m^2^)	16.4 (2.6)	16.3 (2.5)	16.7 (2.9)	16.8 (3.1)	16.4 (2.6)	0.087
Waist circumference (cm)	57.5 (7.8)	57.6 (7.3)	58.9 (8.8)	59.7 (9.6)	57.7 (7.9)	0.001
Overweight/obesity* (%)	207 (8.8)	43 (11.1)	26 (13.5)	26 (15.7)	302 (9.7)	0.005
Central obesity* (%)	101 (4.4)	27 (7.3)	11 (6.2)	17 (11.5)	156 (5.3)	<0.001
UPF intake (g/day)	0.0 (0.0)	23.0 (11.4)	66.2 (13.7)	175.3 (80.5)	15.8 (44.6)	<0.001
Energy intake (kcal/day)	1726 (545)	1662 (508)	1736 (531)	1834 (604)	1724 (543)	0.005
Fat intake (g/day)	48.9 (29.3)	54.8 (28.7)	61.7 (28.4)	68.7 (31.8)	51.4 (29.8)	<0.001
Protein intake (g/day)	51.6 (19.2)	52.2 (18.8)	55.7 (18.5)	60.0 (24.0)	52.3 (19.5)	<0.001
Carbohydrate intake (g/day)	270 (97)	240 (84)	239 (90)	243 (87)	263 (95)	<0.001
Modern pattern score	−0.3 (0.6)	−0.0 (0.7)	0.5 (0.9)	1.4 (1.3)	−0.2 (0.8)	<0.001
Traditional dietary pattern score	−0.0 (0.9)	−0.1 (0.9)	−0.1 (0.8)	−0.2 (0.9)	−0.1 (0.9)	0.245
Fruit Intake (g/day)	17.2 (64.2)	31.2 (87.6)	60.3 (102.3)	80.3 (130.6)	24.8 (76.6)	<0.001
Fresh vegetable intake (g/day)	219 (181)	200 (251)	170 (165)	160 (119)	210 (188)	<0.001
Physical activity (MET)	7.4 (15.8)	8.8 (21.1)	8.7 (17.8)	13.0 (21.1)	8.0 (17.0)	<0.001
Alcohol drinking (%)						
No	354 (97.5)	46 (95.8)	31 (96.9)	21 (95.5)	452 (97.2)	0.867
Yes	9 (2.5)	2 (4.2)	1 (3.1)	1 (4.5)	13 (2.8)	
Non‐smoker (%)	354 (100.0)	47 (100.0)	28 (100.0)	21 (100.0)	450 (100.0)	
Hours of sleep/day (%)						
<6	0 (0.0)	0 (0.0)	2 (2.0)	0 (0.0)	2 (0.2)	
6–9	384 (54.9)	81 (60.4)	55 (55.0)	69 (62.7)	589 (56.4)	0.001
>9	316 (45.1)	53 (39.6)	43 (43.0)	41 (37.3)	453 (43.4)	

*Note*: Numbers in the table: *n* (%) for categorical variables and mean (SD) for continuous variables; Overweight/obesity defined by age‐ and sex‐specific BMI and waist circumference cut‐offs.^21^, ^22^

### 3.3 Overweight/obesity status by UPF intake

**FIGURE 3:**
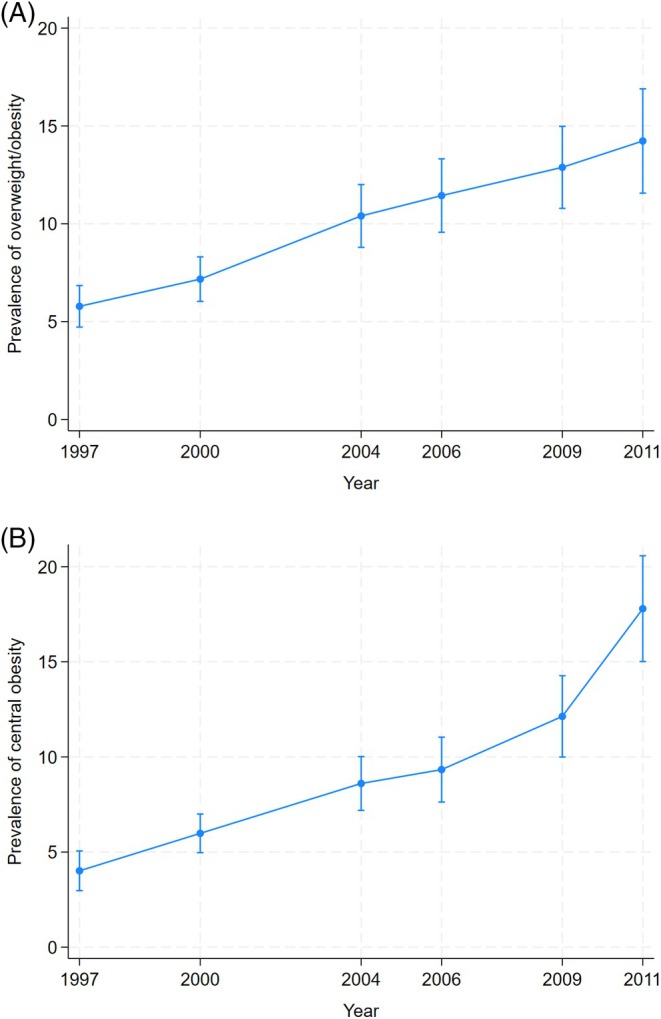
Age‐ and sex‐adjusted prevalence of overweight/obesity* in children and adolescents attending CHNS in 1997–2011. *Overweight/obesity and central obesity defined by age‐ and sex‐specific BMI (A) and waist circumference (B)^21^, ^22^ respectively. Prevalence is estimated from logistic regression adjusted for age and sex.

During the study period, the age‐ and sex‐adjusted mean BMI in children and adolescents increased from 17.3 kg/m^2^ in 1997 to 18.0 kg/m^2^ in 2011. An increase was also observed for mean WC from 61.3 cm in 1997 to 64.2 cm in 2011. The age‐ and sex‐adjusted prevalence of overweight/obesity measured by BMI in the same period increased from 5.7% to 14.2% (Figure 3A), while central overweight/obesity measured by WC increased from 4.0% to 17.8% (Figure 3B).

At baseline, the mean BMI for children and adolescents having UPF consumption levels of 0, 1–49, 50–99 and ≥100 g/day was 16.4 kg/m^2^, 16.3, 16.7 and 16.8 kg/m^2^, respectively, while the WC was 57.5 cm, 57.6, 58.9 and 59.7 cm (*p* = 0.001). The prevalence of overweight/obesity defined by BMI was higher among those with UPF consumption ranging from 8.8% for no consumers, increasing to 11.1% for 1–49 g/day, 13.5% for 50–99 g/day and to 15.7% for ≥100 g/day (*p* = 0.005), with a similar pattern for central obesity defined by WC, with the prevalence from 4.4% for none to 11.5% for ≥100 g/day (*p* < 0.001) (Table 1).

### 3.4 The association between UPF consumption and overweight/obesity

Table 2 shows the association between overweight/obesity and UPF consumption level from mixed effect analysis.

**TABLE 2:** Odds ratio (95% CI) for overweight/obesity by UPF intake levels among children and adolescents aged 6–18 years attending CHNS in 1997–2011.

	UPF intake (g/day)				Trend *p*‐value
	None	1–49	50–99	≥100	
Overweight/obesity					
Unadjusted	1.00	1.12 (0.83–1.52)	1.89 (1.24–2.88)	2.60 (1.55–4.38)	<0.001
Model 1	1.00	1.70 (1.25–2.32)	2.47 (1.63–3.77)	2.70 (1.61–4.53)	<0.001
Model 2	1.00	1.48 (1.04–2.10)	2.52 (1.59–3.99)	2.21 (1.24–3.92)	<0.001
Model 3	1.00	1.38 (0.98–1.94)	2.01 (1.25–3.24)	1.53 (0.82–2.86)	0.013
Central obesity					
Unadjusted	1.00	2.80 (2.10–3.73)	3.77 (2.54–5.61)	4.45 (2.75–7.21)	<0.001
Model 1	1.00	2.25 (1.66–3.06)	3.37 (2.21–5.13)	4.52 (2.70–7.56)	<0.001
Model 2	1.00	2.11 (1.50–2.98)	3.13 (1.96–4.99)	4.12 (2.35–7.24)	<0.001
Model 3	1.00	1.84 (1.30–2.60)	2.13 (1.30–3.48)	2.15 (1.14–4.05)	<0.001

*Note*: Overweight/obesity defined by age‐ and sex‐specific BMI and waist circumference cut‐offs.^21^, ^22^ OR (95% CI) from mixed effect logistic regression analysis. Model 1 adjusted for age, sex and energy intake. Model 2 further adjusted for intake of fat, income, education, residence (urban/rural) and physical activity. Model 3 further adjusted for dietary patterns (traditional pattern characterized by high intake of rice, pork and vegetables, and low intake of wheat; a modern dietary pattern had high intake of fruit, soy milk, egg, milk and deep‐fried products). All the adjusted variables (except sex) are treated as time‐varying covariates.

The unadjusted odds ratios (ORs) (95% CIs) of overweight/obesity defined by BMI were 1, 1.12 (0.83–1.52), 1.89 (1.24–2.88), 2.60 (1.55–4.38) and 2.60 (1.55–4.38) respectively, for UPF consumption level of none, 1–49, 50–99 and ≥ 100 g/d. The ORs were not changed substantially when firstly adjusted for age, sex and energy intake (Model 1), either when adjusted for intake of fat, household income, education, residential areas and physical activity in Model 2, or when further adjusted for dietary pattern in Model 3. The adjusted ORs (95% CIs) were 1.38 (0.98–1.94) for UPF intake of 1–49 g/d, 2.01 (1.25–3.24) for 50–99 g/day and 1.53 (0.82–2.86) for ≥100 g/day.

The odds for central obesity increased by 84% (aOR 1.84, 95% CI 1.30–2.60) for UPF consumption of 1–49 g/d, and more than doubled for the consumption of 50–99 g/day (aOR 2.13, 95% CI 1.30–2.48) and ≥ 100 g/day (aOR 2.15, 95% CI 1.14–4.05) after the adjustment of the socio‐demographic and behavioural factors.

The association between overweight/obesity defined by either BMI or WC and UPF was not different within subgroups by age, sex and residence (Tables 3A and 3B).

**TABLE 3A:** Subgroup analyses of the association between UPF intake and overweight/obesity.^a^

	UPF intake (g/day)				*p* for trend	*p* for interaction
	None	1–49	50–99	≥100		
Sex						0.100
Male (*n* = 1839)	1.00	1.70 (1.07–2.70)	3.48 (1.92–6.30)	1.79 (0.84–3.81)	<0.001	
Female (*n* = 1598)	1.00	1.19 (0.69–2.06)	1.66 (0.78–3.51)	3.14 (1.27–7.81)	0.017	
Residence						0.905
Rural (*n* = 2621)	1.00	1.37 (0. 89–2.10)	2.27 (1.27–4.05)	2.25 (1.07–4.75)	0.002	
Urban (*n* = 861)	1.00	1.89 (0.99–3.57)	3.50 (1. 56–7.84)	2.21 (0.85–5.768)	0.009	
Age (years)						0.789
6–12 (*n* = 2933)	1.00	1.28 (0.99–1.65)	1.79 (1.29–2.46)	1.38 (0.95–2.01)	0.002	
13–18 (*n* = 504)	1.00	1.2 (0.81–1.76)	1.48 (0.86–2.54)	0.97 (0.46–2.06)	0.445	

*Note*: OR (95% CI) from mixed effect logistic regression analysis. Models adjusted for age (not for age subgroup analysis), sex (except for sex subgroup analysis), intake of fat and energy, household income, education, residence (urban/rural) and physical activity. All the adjusted variables (except sex) are treated as time‐varying covariates.

^a^ Overweight/obesity defined by IOTF age‐ and sex‐specific BMI cut‐off.^21^

**TABLE 3B:** Subgroup analyses of the association between UPF intake and central obesity.^a^

	UPF intake (g/day)				*p* for trend	*p* for interaction
	None	1–49	50–99	≥100		
Sex						0.878
Male (*n* = 1839)	1.00	2.18 (1.32–3.62)	2.85 (1.40–5.81)	3.50 (1. 56–7.83)	<0.001	
Female (*n* = 1598)	1.00	1.95 (1.22–3.11)	3.46 (1.86–6.41)	4.99 (2.25–11.07)	<0.001	
Residence						0.732
Rural (*n* = 2621)	1.00	2.06 (1.37–3.10)	2.41 (1.31–4. 45)	3.54 (1.68–7. 45)	<0.001	
Urban (*n* = 861)	1.00	2.37 (1.27–4.41)	4.66 (2.17–10.04)	5.38 (2.18–13.28)	<0.001	
Age (years)						0.064
6–12 (*n* = 2933)	1.00	3.09 (1.82–5.24)	3.43 (1.71–6. 89)	5.92 (2.76–12.68)	<0.001	
13–18 (*n* = 504)	1.00	1.34 (0.87–2.08)	2.07 (1.09–3.92)	2.22 (0.96–5.13)	0.011	

*Note*: OR (95% CI) from mixed effect logistic regression analysis. Models adjusted for age (not for age subgroup analysis), sex (except for sex subgroup analysis), intake of fat and energy, household income, education, residence (urban/rural) and physical activity. All the adjusted variables (except sex) are treated as time‐varying covariates.

^a^ Central obesity defined by age‐ and sex‐specific waist circumference cut‐off.^22^

## 4 DISCUSSION

Among a total of 3437 children and adolescents aged 6–18 years participating in the China Health and Nutrition Survey in 1997–2011, we found the per capita mean UPF consumption increased fourfold and the proportion of UPF intake ≥100 g/day increased dramatically to 40%. The higher UPF intake was associated with twice the increased risk of overweight and obesity.

Our result of the increased trend of UPF consumption among children and adolescents in China is consistent with global trends.^27^ In addition, the trend in these children and adolescents was more obvious than in Chinese adults in the same study period. We speculate that this results from the targeted UPF marketing and sales to children and adolescents in China. In addition, a recent report from 2018 to 2019 highlights the increased accessibility of services delivered by fast food restaurants, street kiosks and cafés and bars^28^ For example, bubble tea, a tea‐based sugar‐sweetened beverage, has become popular among Chinese youth in recent years^29^ and has been associated with overweight/obesity^30^ and mental health.^31^

Beside the environmental changes, other factors may lead to the increased UPF consumption. For example, UPF are inexpensive, ready to eat and have a longer shelf life, making them a time‐saving option for children and adolescents facing increased study pressures, while also offering convenience for parents and caregivers. In addition, children and adolescents are prone to highly palatable UPF that contain artificial or chemical additives.^32^ It has been reported that UPF consumption is positively associated with a preference for non‐core foods, high in sugar and fat^13^, ^33^ and food fuzziness^13^ in children, which could impact dietary behaviour in later years.

In this sample of children and adolescents, higher UPF consumption was consistently and dose‐responsively associated with an increased risk of overweight and obesity, as defined by either BMI or WC. The robust estimate of the association was in line with other longitudinal studies among similar age populations from European and American countries.^10^, ^11^, ^12^, ^13^ The results of this study and others are also supported by the study in the Netherlands showing that the consumption of minimal unprocessed food (higher in natural saturated fats) has increased the high‐density lipoprotein (HDL) (good cholesterol) level in children.^34^ Our study further indicated that the association of UPF with central obesity was more responsive than overweight/obesity defined by BMI, suggesting visceral fat (harmful fat) is more sensitive for fatness than BMI, which is unable to distinguish the proportion of weight due to fat or muscle. In addition, the association between UPF and obesity was independent of dietary energy intake, suggesting other added non‐nutritional bioactive compounds may contribute to it. For example, some additives such as artificial sweeteners, emulsifiers, thickening and stabilizing agents and bisphenols may have impacts on body weight through pathways of insulin response, or either gut microbiota adipocyte function, or both.^35^

Our results among Chinese children and adolescents underscore the need for further exploration of the impact of the environment and food policy and regulations on unhealthy dietary habits. For example, schools, communities and families could provide a healthy environment and foster healthy dietary behaviours in this population. Childhood and adolescence are critical life stages in which lifelong healthy lifestyle behaviours are shaped and cultivated, as Bandura depicts that there is a reciprocal deterministic relationship between the individual, his/her environment and behaviour.^36^ Timely and adaptive environmental strategies to promote healthy dietary behaviours among children and adolescents are essential, particularly in the context of the dynamic social and economic development in China. These strategies should complement existing lifestyle interventions aimed at combating overweight and obesity in Chinese children and adolescents.^37^, ^38^

This is the first investigation addressing the association between UPF consumption and overweight/obesity among Chinese children and adolescents from the national surveys. The methodological strengths of this study ensured the robustness of the results. For example, anthropometric measures such as height, weight and WC were measured by trained research staff with standardized procedures. Overweight and obesity were defined by both BMI and WC using international age‐ and sex‐specific cut‐offs for cross‐country comparison. The food intake was collected using the 24 h‐recall method supplemented by household inventory record, which was inclusive and completed representing a robust estimate of long‐term habitual intake. The energy and food intake from the surveys have been validated by our previous investigation based on basal metabolic rate.^39^ We used repeated measurements of UPF intake, BMI and WC in the mixed effect analysis to accommodate the within‐person variations during the study period and to minimize the long‐term fluctuation. A series of confounding factors including socio‐demographic, behavioural, health and dietary factors were adjusted, but residual confounding is possible.

Limitations should be noted. Firstly, misclassification was a possibility due to incomplete records on food processing methods in the CHNS survey, which was not specifically designed for NOVA classification. Secondly, some food items (e.g., milky tea) could only be roughly grouped, and the association could be biased due to the complexity of food processing and the variabilities in additive composition between brands for a similar type of product. Thirdly, our sample of children and adolescents was those residing with parents at the time of the surveys, and those attending boarding schools were not included in the sample/study cohort. In China, the proportion of school‐aged children and adolescents at boarding schools has increased over the years to 26.6% in 2011, as reported by the Chinese Department of Education.^40^ This could partly explain the decreased sample size in this study during 1997–2011. A well‐designed school‐based survey is warranted for comprehensive assessment, causality and mechanisms investigations. Biochemical markers such as blood lipid profiles could be included. Finally, residual confounding was still possible due to the lack of data on ethnicity, which is closely related to culinary culture in China.

In conclusion, the mean daily consumption of UPF among Chinese children and adolescents increased four times, reaching 60 g in 2011 across all socio‐economic strata. Higher UPF consumption doubled the risk of overweight/obesity compared to no consumption. Further research is needed to confirm the results of this observational study and to understand the possible mechanisms of UPF's effect on obesity risk.

## AUTHOR CONTRIBUTIONS

ML and ZS conceived and planned the study. ML drafted the manuscript. ZS obtained the data and analysed the data. All authors reviewed the paper and approved the submitted and published versions.

## CONFLICT OF INTEREST STATEMENT

No conflict of interest was declared.
